# The Role of Macrophages During Mammalian Tissue Remodeling and Regeneration Under Infectious and Non-Infectious Conditions

**DOI:** 10.3389/fimmu.2021.707856

**Published:** 2021-07-14

**Authors:** Candice Bohaud, Matt D. Johansen, Christian Jorgensen, Laurent Kremer, Natacha Ipseiz, Farida Djouad

**Affiliations:** ^1^ IRMB, Univ Montpellier, INSERM, Montpellier, France; ^2^ Centre National de la Recherche Scientifique UMR 9004, Institut de Recherche en Infectiologie de Montpellier (IRIM), Université de Montpellier, Montpellier, France; ^3^ Centre for Inflammation, Centenary Institute and University of Technology Sydney, Faculty of Science, Sydney, NSW, Australia; ^4^ Clinical Immunology and Osteoarticular Diseases Therapeutic Unit, Department of Rheumatology, Lapeyronie University Hospital, Montpellier, France; ^5^ INSERM, IRIM, Montpellier, France; ^6^ Systems Immunity Research Institute, Heath Park, Cardiff University, Cardiff, United Kingdom

**Keywords:** mammals, regeneration, repair, macrophages, infectious conditions, non-infectious conditions

## Abstract

Several infectious pathologies in humans, such as tuberculosis or SARS-CoV-2, are responsible for tissue or lung damage, requiring regeneration. The regenerative capacity of adult mammals is limited to few organs. Critical injuries of non-regenerative organs trigger a repair process that leads to a definitive architectural and functional disruption, while superficial wounds result in scar formation. Tissue lesions in mammals, commonly studied under non-infectious conditions, trigger cell death at the site of the injury, as well as the production of danger signals favouring the massive recruitment of immune cells, particularly macrophages. Macrophages are also of paramount importance in infected injuries, characterized by the presence of pathogenic microorganisms, where they must respond to both infection and tissue damage. In this review, we compare the processes implicated in the tissue repair of non-infected *versus* infected injuries of two organs, the skeletal muscles and the lungs, focusing on the primary role of macrophages. We discuss also the negative impact of infection on the macrophage responses and the possible routes of investigation for new regenerative therapies to improve the recovery state as seen with COVID-19 patients.

## Introduction

Most mammals, such as mice and humans, possess limited regenerative capacities. Only a few rare tissues or organs such as muscle, lung epithelium and liver can regenerate in adult mammals after ablation or injury, leading to an integrated morphological and functional structure. For non-regenerative adult organs, while critical injuries usually lead to a definitive disruption of tissue architecture and functionality, superficial wounds are often followed by tissue remodelling and scar formation (1, 2). During the early stages of embryonic development, mammals develop an extraordinary regenerative potential, which is rapidly lost when reaching the adult stage. For instance, E10 mouse embryos completely regenerate their forelimb bud after ablation (3), supporting the view of a regenerative capacity loss during development rather than an intrinsic mammalian deficiency. Therefore, it may be perceived that mammalian species possess the full capacity to regenerate entire parts of their body, as newts, but this potential is progressively lost during their development. Elucidation of the regeneration mechanisms related to embryos and adult mammals still requires extensive studies in order to propose and develop novel therapies aimed at restoring tissues and organs in humans.

Tissue lesions and their repair/regeneration process in mammals are commonly studied under so-called non-infectious conditions. Non-infected injuries include the generation of pathogen-free lesions, such as sterile amputation, burn, freezing, crushing or drug toxicity (4–6). In this context, many key mechanisms for the regeneration of tissues and organs have been identified, including cell death at the site of the injury as well as danger signals, favouring massive recruitment of the immune cells, including macrophages (MФ). In contrast, infected injuries are characterized by the presence of pathogenic microorganisms and include open wounds contaminated with external infectious agents or tissue/organ alterations caused by systemic or localized infections already present. Under these conditions, MФ must respond to the two types of dangers related to infection and tissue damage.

While studies on regeneration are usually performed under non-sterile conditions, they do not reflect most of the natural situations and, therefore, fail to recapitulate all the complexities of the interactions encountered in wounds. Thus, to improve our knowledge and further develop protocols for the treatment of injuries in mammalians, a comprehensive comparison of the processes implicated in the tissue repair of non-infected *versus* infected injuries requires additional investigations to identify critical pathways involved in tissue regeneration. In this review, we will mainly focus and compare the role of MФ in regeneration of non-infected *versus* infected injured tissue in mammals. We will discuss the negative impact of infection on the MФ responses, in turn altering the response and the fate of proliferating precursor cells. Considering the specificity of the processes involved in the regeneration and the broad spectrum of MФ phenotypes in each regenerating organ, we will exclusively focus our review on two tissues, the skeletal muscles directly exposed to non-infected and infected open wounds and the lungs, which are continuously exposed to potential contaminated air.

## Definition of Regeneration and Role of Inflammation in Non-Infected Versus Infected Injuries

Regeneration is the process that leads to the restoration of a tissue or an organ following injury or amputation in terms of mass, structure and functions. In contrast, tissue repair, distinct from regeneration, is the most common mechanism occurring after a major injury in adult mammals. It does not allow the tissue to recover its original architecture due to the formation of a fibrotic scar, resulting in the altered functionality and motility of the repaired tissue/organ.

Non-infected injuries are as diverse as the organs they can affect: burn, crush, cut, drug exposure. Despite their vast intrinsic nature, all injuries induce the same course of events that include wound closure, recruitment of immune cells and an acute inflammation phase, death of the damaged cells followed by resolution of the inflammation, cell de-differentiation and proliferation, disappearance of immune cells and formation of a novel tissue/organ (7). During the acute inflammation phase at the site of injury, necrosis leads to the rapid death of damaged cells and is characterised by the sudden rupture of the cell membrane and the release of danger molecules designated Damage-Associated Molecular Patterns (DAMPs) in the surrounding environment (8, 9). DAMPs are intracellular components, such as DNA, RNA, proteins or the vast group of alarmins like High Mobility Group Box 1 (HMGB1) (10), IL-1α (11) and IL-33 (12, 13). The release of DAMPs triggers acute inflammation as well as the recruitment of immune cells to the site of injury.

Infected injuries are characterized by the presence of microbes or in many cases, opportunistic or pathogenic organisms. In addition to the traditional DAMPs released by the necrotic tissue, the presence of pathogens induces the recognition of Pathogen-Associated Molecular Patterns (PAMPs), ascribed as molecules located on the surface of the pathogens, which activate the immune response to the invading pathogen (14). PAMPs are a diverse group of signalling molecules that include lipopolysaccharide (LPS), single- and double-stranded viral RNA, flagellin and peptidoglycan, and are recognized by the Pattern Recognition Receptors (PRRs) located at the surface of nearby immune cells (15). The recognition of PAMPs activates pro-inflammatory pathways, ultimately leading to acute inflammation.

Although their mechanisms of action appear strictly different, inflammation in both non-infected and infected injuries appears to be induced by the same early pattern of immune cells recruitment. Indeed, within the first hours following injury, neutrophils enter the insulted site to immobilise, kill and phagocyte the pathogens or clear cellular debris (16–20). Neutrophils assist the recruitment of blood-derived monocytes *via* the secretion of the chemoattractant LL-37, cathepsin G, alpha defensin and azurocidin (21–23), which then differentiate into MФ. After a few hours or a few days, B cells and T cells from the adaptive immune system enter the scene, providing a sustained systemic immunity until pathogen clearance is achieved (24). Injury and infection resolution is associated with a switch from a pro-inflammatory to anti-inflammatory/pro-resolving microenvironment and then, immune cells disappear progressively, either leaving the injured site or dying in the regenerating tissue (20, 25–27). Each of these steps is essential to ensure full tissue restoration by avoiding establishment of a chronic inflammation at the wound site that would block the subsequent regeneration process (28). While the role of the innate and adaptive immune response in resolving inflammation and regeneration has been extensively described, we will mainly focus on the innate immune response, and more specifically on MФ, in these processes.

## Role of Macrophages During Injury

Monocyte-derived MФ and tissue resident MФ are innate immune cells present in all tissues during the entire life (29). MФ are heterogenous and highly plastic cells (30). This plasticity allows them to adapt to their environment and to exert various functions within the tissues. At steady state, resident MФ patrol within the tissues to maintain homeostasis by clearing dead cells, promoting cellular communication and phagocytosing invading microorganisms. In mice, tissue resident MФ have a pre-natal origin (yolk sac- and foetal liver-derived) and are believed to mostly maintain themselves by self-proliferation (31). Their function, localisation, origin and phenotypic traits are dependent on the expression of specific transcription factors. Many review articles have summarised the current knowledge of tissue resident MФ development and function (32–35). In the event of an injury or during pathogen invasion, tissue resident MФ are activated and assist the recruitment of neutrophils (36–38) and monocyte-derived MФ (36, 39). The infiltrating MФ can be roughly classified into two bulk populations: the pro-inflammatory MФ, recruited within two days following the initial insult, and the anti-inflammatory MФ, appearing usually from day three. Based on their secretome profile, MФ were initially classified as “M1” and “M2”, albeit it is now clear that their diversity is much more complex than originally suspected, further segregating into additional subpopulations (40, 41). Appearing first at the site of injury, the pro-inflammatory MФ help at eliminating dead neutrophils, phagocytosed pathogens or dying cells from the injured area (33). In contrast, anti-inflammatory MФ, emerging essentially during the second wave of MФ recruitment, are considered as pro-resolving and are essential for the recruitment of new progenitor cells and for the resolution of the inflammation (42). The anti-inflammatory MФ are either derived from the pro-inflammatory MФ pool, which undergo a phenotypic switch (43) notably after phagocytosis of the dead neutrophils (25, 44) or are directly recruited from blood monocytes (45). MФ are key players at every step of tissue injury resolution. Not only critical for controlling inflammation, they also intervene during parenchymal and mesenchymal repair processes as well as in fibrosis (46). Through the secretion of various factors [Transforming Growth factor beta (TGF-β), Platelet-derived growth factor (PDGF), Vascular endothelial growth factor (VEGF), Tumour necrosis factor (TNF), Interleukin 1 (IL-1) and matrix metalloproteinases (MMPs)], MФ promote angiogenesis, recruit fibroblasts and keratinocytes and participate to the remodelling of the extracellular matrix. The elimination of murine MФ, consecutive of the injection of lipochlodronate, negatively impacts wound healing and collagen deposition, translating into the loss of tissue functionality. The temporal depletion of MФ during the pro-inflammatory phase or during the resolution phase of inflammation has a differential impact on tissue restoration, thus inferring that MФ subtypes play different roles during this process. Time and spatial regulation of MФ accumulation and polarization is equally important during pathogen infection (47). Strong evidence indicates that the clearance of pathogens colonizing the injured area requires MФ which are essential to the healing process (48, 49) although many important questions remain unaddressed. For instance, it is not known whether the pre-established pathogen-induced inflammation detrimentally impacts the speed and efficiency of tissue repair after tissue injury. Additionally, it remains also to be established whether the cumulative presence of DAMPs and PAMPs affects resolution of the injury in an organ-dependent manner.

## Skeletal Muscle Regeneration

Skeletal muscles which are composed of myofibers, connective tissue, nerves, blood, lymph vessels and immune cells with tissue resident MФ, represent approximately 40% of the body mass in humans. This organ can fully regenerate after minor injury (50, 51). However, severe injuries such as mechanical shock, burn and deep laceration can lead to incomplete healing, scar formation and fibrosis, resulting in a long-lasting or permanent loss of function.

Muscle repair is a complex process. Damaged myofibers and endothelial cells first undergo a necrosis/degeneration stage, characterized by the release of DAMPs and triggering acute inflammation. Then, the quiescent muscle stem cells adopt an activated state, proliferate and differentiate, providing precursors cells, which will ultimately lead to mature muscle fibers (5).

Muscle stem cells or satellite cells play an important role in tissue restoration after muscle injury. These cells, expressing in resting states various specific markers including the paired homeobox factors Pax3 and Pax7, reside in specialized local environment between the basal lamina and the myofiber sarcolemma (52). When muscle damage is induced, these quiescent cells rapidly transit to an activated state characterized by the expression of markers such as the myogenic regulatory factors MYF5, MYOD, MYOGENIN, MRF4 to regenerate the injured tissue (53). After their activation, satellite cells proliferate. While one part of the proliferating cells differentiates into myoblasts to regenerate the damaged muscle, another part of the cells reconstitutes the pool of quiescent satellite cells. The newly formed myoblasts can then fuse with the pre-existing myoblasts in the tissues or fuse with each other through the expression of several factors, such as transforming growth factor beta (TGFβ) to repair damaged muscle fibers (54–56). This complex mechanism involving cell migration, recognition of the ongoing events and cell adhesion is not fully understood (54, 55). This cascade of events, relies on immune cell response and more particularly on the MФ response. Indeed, muscle resident MФ are mostly located in the perimysium (connective tissue surrounding muscle fascicles) and epimysium (fascia surrounding the muscle) and are estimated to be at a ratio of one MФ per five muscle fibers. However, both the origin and role of the muscle resident MФ in development and tissue regeneration remain poorly described (57–60).

**Figure 1:**
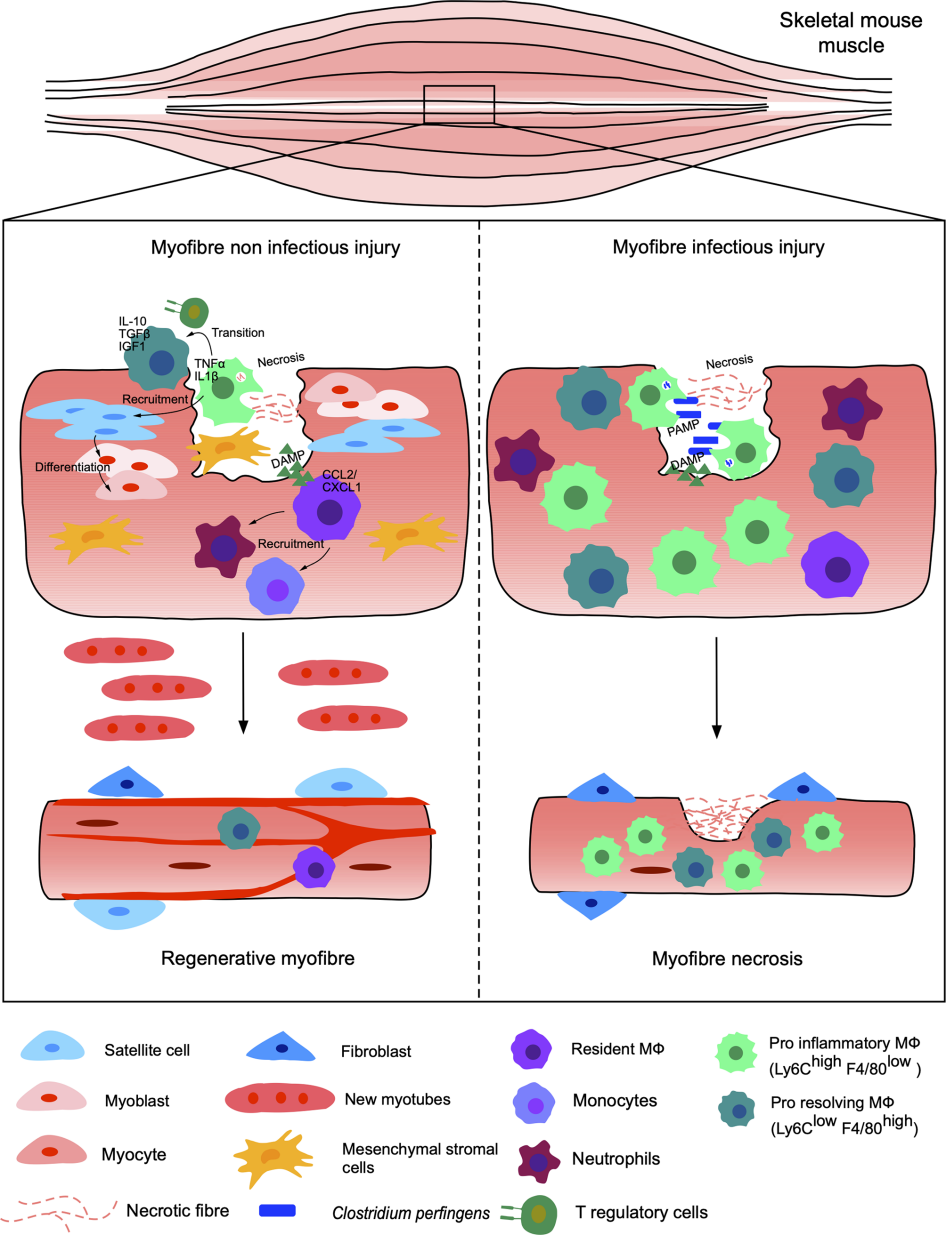
Non-infected versus infected injuries of the murine skeletal muscle. Injury of muscle myofibers under sterile conditions, such as freezing, chemical injections or exposure to toxins, leads to necrosis of the myofibers and endothelial cells responsible for the release of DAMPs. Resident MФ respond to DAMPs and recruit neutrophils and other monocyte-derived MФ by releasing CCL2/CXCL1. The pro-inflammatory Ly6Chi F4/80 low MФ, TNFα and IL-1β positive, eliminate dead cells and recruit satellite stem cells as well as myogenic precursors secreting pro-inflammatory cytokines. The pro-inflammatory MФ are then gradually replaced by anti-inflammatory MФ, known as pro-resolving Ly6Clow F4/80 high secreting IGF1, IL10 and TGFβ. The switch of MФ subpopulations is induced in part by the phagocytosis of debris by pro-inflammatory MФ, which activate the AMPK and C/EBPb pathways, but also *via the* presence of T regulatory cells. Finally, stromal mesenchymal cells (FAP) are recruited and support myogenesis. The satellite cells generate myoblasts, differentiating into myocytes giving new myofibers and functional regenerated tissue. This same injury under infectious conditions, for example in the presence of *Clostridium perfringens*, leads to a significant recruitment of pro-inflammatory MФ. The pro- and anti-inflammatory balance is dysregulated and the inflammation becomes excessive and persistent. Thus, the tissue does not regenerate and becomes necrotic, losing its functional properties.

Currently, most studies concentrate on the resolution of injury under non-infectious conditions, while most open injuries occur under non-sterile conditions and exposed to microbes, eventually leading to complicated clinical manifestations and diseases (61). Thus, understanding the mechanisms regulating infected *versus* non-infected skeletal muscle injury regeneration are of particular importance to pinpoint the key elements necessary for restoration of functional tissues/organs (**Figure 1**).

### Non-Infected Skeletal Muscle Injury

Regeneration of skeletal muscle in mice is usually studied after injury of the Tibialis anterior, *Triceps brachii*, Gastrocnemius and Soleus muscles (4, 5). Muscles are usually injured either by freezing or following injection of chemicals [barium chloride (BaCl_2_)] or toxins from snake venom [notexin (NTX) and cardiotoxin (CTX)] (6). CTX offers the best regeneration outcome, based on histological analyses (6). However, the use of CTX to measure the wide array of tissue repair and regeneration processes need to be carefully considered within the context of the mechanisms required at the cellular level.

MФ are present and essential in every single step required for the regeneration process since their depletion inhibits tissue restoration (43, 62–65). This has been shown by depleting macrophages *via* different pharmacological or genetic approaches, including (i) lipochlodronate injection, (ii) the use of transgenic mice with the CD11b promoter directing the expression of a diphtheria toxin receptor (43) or (iii) the use of CCL2- and CCR2-knockout mice (66, 67).

Upon injury, resident MФ do not phagocyte the dying cells, but rapidly respond to DAMPs and promote the recruitment of other leukocytes, such as neutrophils and monocytes, notably *via* the secretion of CCL2 and CXCL1 (68, 69). Within several hours following injury, neutrophils followed by Ly6C^high^ F4/80^low^ monocyte/MФ enter the injured site. These pro-inflammatory MФ clear the dead cells, promote the recruitment of satellite cells and myogenic precursor cell proliferation *via* secretion of IL-6, TNF, G-CSF and IL-1β, while simultaneously blocking their differentiation (70–74). From 48 hrs after injury, the pro-inflammatory Ly6C^high^ F4/80^low^ monocytes/MФ progressively transition to “pro-resolving” Ly6C^low^ F4/80^high^ MФ, producing high levels of insulin-like growth factor-1 (IGF-1), IL-10 and TGFβ-1, dampening the inflammatory response and favouring tissue repair (75–78). The phenotype skewing of infiltrating MФ is, at least partially, due to phagocytosis of debris, which activates the AMPKα1 and C/EBPβ pathway and results in the expression of typical anti-inflammatory genes (78, 79). This phenotypic switch is also dependent on Treg-induced Interferon gamma (IFN-γ) reduction (80). In parallel, from 1 day post injury, Lin^−^ Integrin-α7^−^ Sca1^+^ PDGFR-α^+^ fibro/adipogenic progenitors (FAP) and non-myogenic mesenchymal cells, are recruited to the injured site to support myogenesis (81). Although essential, FAP expansion is transient and must be rapidly reduced under normal conditions of regeneration. It can become persistent in degenerative conditions, such as chronic lesions and muscular dystrophies. Inflammatory MФ participate in the programmed elimination of these progenitors by induction of apoptosis mediated by TNF (82). The interaction between cell progenitors and inflammatory cells in the damaged muscle influences the course of the regeneration process. However, the macrophage phenotype can also be influenced by the extracellular environment. An infectious environment, described in the following section, can modulate the response of these cells and impair regeneration of muscle tissues.

### Infected Skeletal Muscle Injury

Muscle repair of an infected injury has received little attention, but studies agree that the presence of bacteria or parasites can substantially delay muscle regeneration, often resulting in a loss of the muscle mass and overall mobility and function (83).

Muscle infection by the Gram-positive anaerobic bacteria, *Clostridium perfringens* is one of the major causes of gas gangrene development, also called clostridial myonecrosis. Patients with peripheral vascular disease and type I diabetes are more prone to gas gangrene, however the common sources are traumatic lesion and deep surgery. Fatal if left untreated, gas gangrene is estimated to affect at least 1000 patients in the USA every year, but the disease burden in less developed countries remains unknown (84, 85). A mouse model of gas gangrene, consisting of intramuscular injection of *C. perfringens*, results in increased inflammation, dysregulated recruitment and maintenance of both pro- and anti-inflammatory MФ with deficient muscle regeneration, compared to non-infected acute muscle injury (61).

Skeletal muscle can also be infected by the parasite *Toxoplasma gondii*, which by itself induces chronic inflammation and long-term muscle damage, myositis and cachexia, characterized by 20% body mass loss and elevated TNF, IL-1 and IL-6 (86, 87). A comparison between non-infected injured and *T. gondii*-infected injured muscles revealed that *T. gondii* led to the accumulation of pro-inflammatory MФ after injury and impaired regeneration (86). The lack of pro-inflammatory to pro-resolving MФ switch is likely resulting from a dysregulation of T cells since Tregs have been shown to be required for M1 to M2 transition (86). Single cell RNA sequencing (scRNA-seq) confirmed striking differences in the transcriptomic profiles of MФ recruited after injury in infected *versus* non-infected mice, with a prevalence of pro-inflammatory phenotypes in the infected injured mice (88). However, many aspects of skeletal muscle repair during *T. gondii* infection remain to be understood, requiring further investigations.

## Lung Regeneration

The lung is an essential physiological mammalian structure responsible for gas exchange. It is a highly dynamic micro-environment which plays a critical role in cellular respiration and in mounting an immunological response to both infectious agents such as bacteria and viruses, and non-infectious agents such as environmental pollutants. Accordingly, the lung possesses a high propensity to regenerate through widespread proliferation and differentiation of progenitor cells upon injury. Epithelial insults and/or respiratory infections can lead to the disruption of gas exchange at the alveoli, destroying alveolar epithelial type 1 (AT1) and type 2 (AT2) populations, which in extreme cases can lead to acute respiratory distress syndrome (ARDS).

The lung airway is a complex architecture composed of a large diversity of cells with dedicated functions, depending on where they stand in the lungs (89, 90). The epithelium of the trachea and bronchioles consists mainly of basal cells, club cells, goblet cells and ciliated cells (91). Basal cells in humans are distributed in the trachea to the terminal bronchioles and are mainly found in the trachea of mice. The renewal rate of these cells in physiological conditions is low, but after injury, they can differentiate into secretory cells, goblets and multiciliated cells (92–95). Club cells, which are abundant in the murine bronchioles can also differentiate into ciliated cells after injury. Goblet cells are found within the respiratory and intestinal epithelial lining, and are responsible for mucin production, commonly characterized by MUC5AC and MUC5B production. The final structure of the bronchial tree, the alveoli, comprises two types of epithelial cells: type I alveolar (AT1) and type II alveolar (AT2) cells (96). AT2 cells make up only 5% of the alveolar surface, but these can differentiate into AT1 after injury to maintain the integrity of the epithelium (89, 97). In addition, once differentiated, these cells are versatile and can return, at least partially, to a precursor/undifferentiated phenotype. At steady state, all these cells are in a quiescent state, which has made their roles and identification very challenging (89, 98–100). Importantly, these cells can undergo proliferation and differentiation in response to various stimuli or injuries (101). However, the type and extend of the injury as well as the regenerative capacity of lungs is highly dependent on the nature of the injury (102–104). As such, careful considerations are needed with regard to the choice of the model used. MФ represent crucial cells for the pulmonary function and particularly in the response to lung injury. A recent study unscored the importance of MФ in the lung epithelium regeneration *via* an IL-33/ST2 mechanism (105). The physiological role of MФ in lung regeneration is still poorly understood and is accentuated by an ongoing debate on the origin, function and phenotypic traits of the different pulmonary MФ subtypes.

At steady state, lung tissues harbour two types of resident MФ: alveolar and interstitial (106). The alveolar MФ, located in the lumen of the alveoli, live in a strategic place because they are in direct contact with the inhaled air and, therefore, represent the first line of defense against invading particles or microbes. They trigger the immune response to dangers while preventing excessive responses and tissue damage. This MФ population also regulates the surfactant homeostasis, which is critical for gas exchange (107). Originated mainly from fetal monocytes, they adopt their phenotype shortly after birth and are dependent on the GM-CSF/PPAR-γ (108, 109) pathway as well as their own production of TGF-β (110). The pulmonary environment, at steady state, gives them an anti-inflammatory phenotype.

Interstitial MФs are located between the pulmonary epithelium and the capillaries, predominantly within the alveolar interstitium, the submucosa and the perivascular adventitia. These cells assist alveolar MФ to protect the tissue against infections. Two distinct subsets of interstitial MФ have recently been identified in murine lungs, including LYVE‐1^low^ MHC Class II^high^ involved in antigen presentation, and LYVE‐1^high^ MHC Class II^low^ specializing in tissue repair (111, 112). However, the origin of the interstitial MФ is complex, as this population are thought to have a mixed origin, which makes their phenotypic characterization challenging. Mostly derived from the bone marrow, a small proportion of interstitial MФ has been shown to originate from the yolk sac (113, 114), but this requires further investigations.

Survival and renewal of the resident lung MФ depend on both the type and the size of injury encountered. While alveolar MФ proliferate slowly to renew themselves at steady state in a manner dependent on M-CSF and GM-CSF (115), severe injury promotes their disappearance. To repopulate lung tissues, these cells can either proliferate locally or be replaced by monocyte-derived MФ, which, over time, take up the alveolar MФ characteristic. Interstitial MФ can also spread near the injured site *via* differentiation of blood, local or splenic monocytes into interstitial lung MФ. Survival of monocyte-derived MФ recruited following injury varies also depending on the nature of the injury (116–118).

### Non-Infected Lung Injury

**Figure 2:**
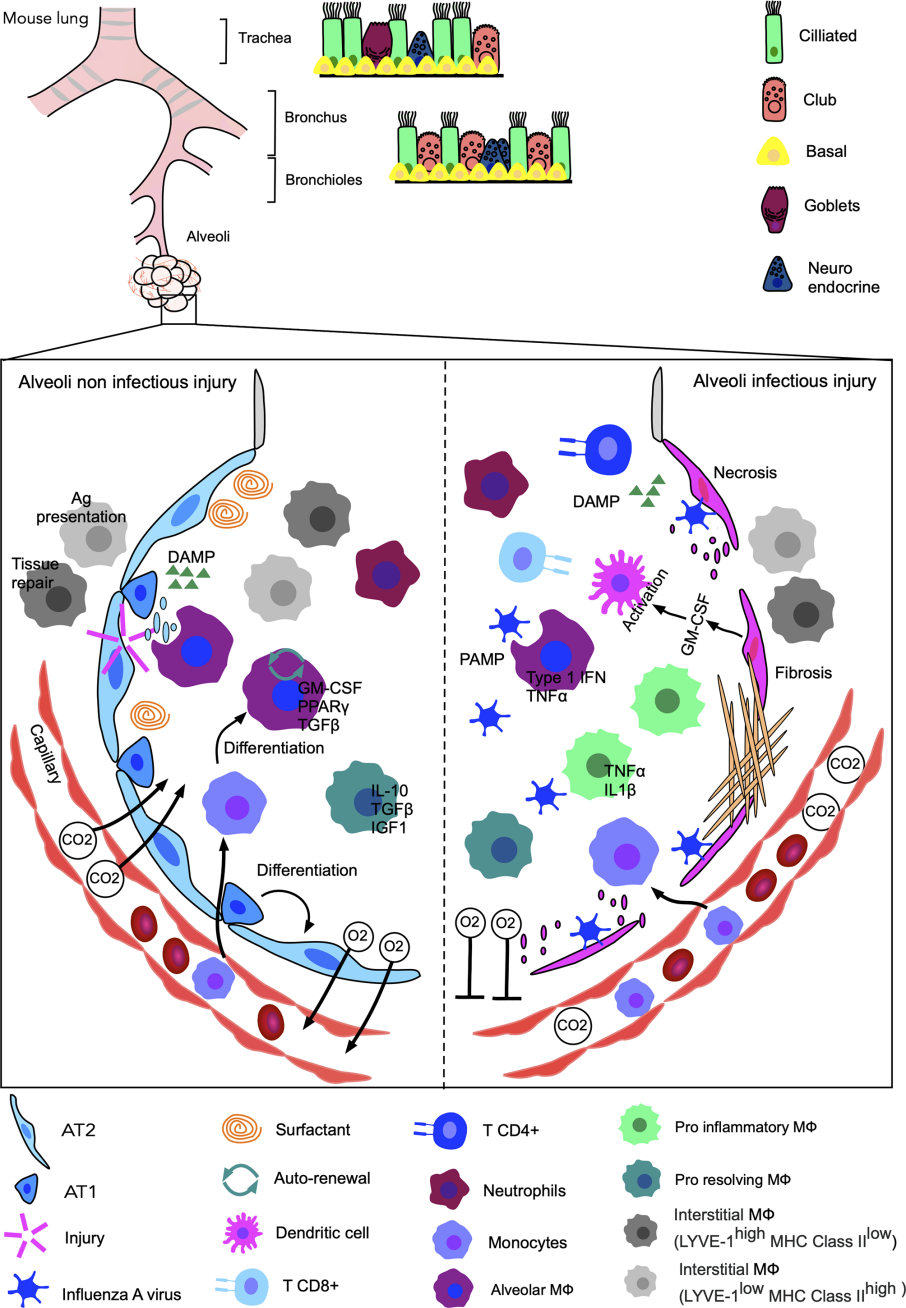
Non-infected versus infected injuries of the murine lungs. The release of DAMPs linked to a sterile induced-injury (ventilator-induced, acid aspiration, contusion, bleomycin injection, resection) in the pulmonary alveolus triggers an immune response consisting primarily of neutrophils and MФ, resulting in regeneration of the pulmonary epithelium. Alveolar MФ (GM-CSF, PPAR-*γ* and TFG-β), the first barrier to danger, phagocytose debris and dead cells. They prevent excessive inflammation, the formation of lesions and regulate surfactant. They can self-renew or be substituted by monocyte-derived MФ. The two types of interstitial MФ, located in part between the pulmonary epithelium and blood capillaries, enter the alveoli and help the alveolar MФ to respond to dangers. LYVE-1^low^ MHC Class II^high^ MФ are responsible for antigen presentation while LYVE-1^high^ MHC Class II^low^ help tissue repair. Upon tissue restoration, exchanges of O_2_ and CO_2_ through the capillaries return to normal. Injury in infectious conditions, for example caused by infection with Influenza A virus in mice, may impair the ability to regenerate. Alveolar MФ phagocytes debris, dead cells and the infectious agent. Alveolar MФ initially anti-inflammatory, adopt an inflammatory phenotype with the release of IFN-*γ* and TNF-α leading to the production of GM-CSF by epithelial cells, in turn activating dendritic cells (DC). Three weeks after infection of MФ, neutrophils and CD4^+^, CD8^+^ T cells are still present. Excessive inflammation with the persistent presence of pro-inflammatory MФ can eventually lead to extensive cell necrosis and the formation of non-functioning fibrous scar tissue. As a result, oxygen is no longer properly transferred to the blood capillaries and breathing difficulties may occur.

Various animal models have been developed in the laboratory to mimic the conditions of non-infectious lung injury in human: ventilator-induced injury (119), acid aspiration (120), contusion (121–123), bleomycin injection (124), exposition to SO_2_ (125), Cl_2_ (126) and cigarette smoke (127, 128) (**Figure 2**). The role of MФ in each of these models has been investigated by focusing predominantly on alveolar MФ. Mechanical ventilation-induced injury activates alveolar MФ and transient depletion of these cells using chlodronate improves pulmonary elastance, while reducing oedema and tissue permeability (129, 130). Acid-induced injury triggers the rapid recruitment of neutrophils and blood-monocyte-derived MФ and the release of microparticles within the bronchoalveolar lavage fluid (BALF). These microparticles seem to induce a pro-inflammatory response in alveolar epithelial cell line MLE-12, but are safely removed by resident alveolar MФ in a non-inflammatory MerTK-dependent manner (131). Initial *in vitro* work performed with human alveolar MФ identified their positive role on Type 2 alveolar epithelial cell proliferation *via* the release of PDGF, IGF-1 and FGF, following incubation with silica (132). A recent study identified their direct influence on epithelial cell proliferation following bleomycin-induced injury in mice in a Wnt-dependent pathway (133), concurring with previous study highlighting the importance of Ly6C^hi^ monocytes and MФ in the development and resolution of fibrosis after bleomycin-induced injury (134). Increased numbers of circulating, interstitial and alveolar MФ are found in the lungs following partial pneumonectomy and CCR2^+^ monocytes are essential for lung regeneration (135). Mice have also the capacity to adapt to recurrent oxidative toxicant exposure such as Cl_2_ and this adaptation seems to be dependent on alveolar MФ through the production of PGE2 and TGFβ (136). The balance between pro-inflammatory and anti-inflammatory MФ is essential to ensure meaningful control of inflammation and tissue repair, although the exact role of the different MФ subsets in the development and resolution of fibrosis is still poorly understood (137). However, the importance of the first pro-inflammatory phase should not be disregarded, as evidence recurrently emphasizes its importance for the initiation of the later pro-resolving phase (138).

### Infected Lung Injury

Most lung injuries following bacterial, fungal, parasitic or viral infection result in a loss of tissue elastance and integrity, often requiring extensive regeneration following elimination of the pathogen (139, 140). However, tissue regeneration can fail, precluding full pulmonary recovery in some patients. Tissue recovery and injury resolution can be very challenging and is highly dependent on the nature of the infectious agents (**Figure 2**).

## Influenza Viruses

Influenza viruses are a family of RNA viruses infecting epithelial cells present in the upper and lower respiratory tract. The infected cells often die *via* apoptosis, leading to extensive infection and epithelial injury. Although most individuals recover well after infection, some patients experience long-term consequences, characterized by alveolar hypersensitivity (alveolitis), acute respiratory distress syndrome (ARDS) and fibrosis (141, 142). Murine models of influenza infection revealed that the inflammatory status in BALF can persist even after the infection is cleared. Importantly, MФ, neutrophils, CD4^+^ and CD8^+^ T cells are present at least three weeks after infection, even after influenza virus is no longer present. Furthermore, alveolitis was observed up to 60 days after infection. In addition, influenza virus not only induces cellular stress but affects also the lungs at the transcriptomic and epigenetic levels, leading to long-terms lung changes as a consequence of infection (143). Resident alveolar MФ are the first immune cells to react to viral infection due to their close proximity to the site of infection. Alveolar MФ actively phagocytose the viral particles as well as the dead and apoptotic cells and produce type I IFN, which are essential for the control of viral infection (144–147). The activated alveolar MФ also release TNF, an important factor inducing GM-CSF production from epithelial cells, which in turn activates CD103^+^ DC and induced AT2 epithelial cell proliferation, required for the regeneration process (148, 149). Following influenza infection, monocyte-derived MФ are recruited to the lungs where they persist and over time, acquire hallmarks of tissue resident alveolar MФ where they participate in the replenishment of the alveolar MФ pool following resolution of acute infection. Interestingly, the monocyte-derived MФ transcriptomic profiles remain altered for several weeks after influenza infection, while tissue resident alveolar MФ are barely affected and, therefore, considered as “terminally sedated” (118, 150, 151). Given the importance of MФ on epithelial cell proliferation and differentiation, it is reasonable to assume that the effectiveness of lung tissue regeneration is dependent on the phenotype of both tissue resident and newly recruited monocyte-derived alveolar MФ.

## Tuberculosis

Tuberculosis (TB), caused by the pathogenic bacteria *Mycobacterium tuberculosis*, is a global health concern and consistently one of the top ten causes of death worldwide (https://www.who.int/tb/publications/global_report/en/). Half of the patients with TB infection present with lung dysfunction, even after chemotherapy or microbiological healing to clearance (152, 153). While the pulmonary epithelium is able to regenerate after injury in some rare cases, this potential is lost in TB patients (152). When the permissive environment for regeneration is altered, a significant deposition of collagen occurs, leading to fibrosis. One of the main causes of this lesion and fibrosis is the formation of structures called granulomas (152). Granulomatous lesions are considered as the hallmark of pulmonary TB and consist of a complex immune structure linking innate and adaptive immunity (154, 155). Following aerosol inhalation, *M. tuberculosis* is rapidly phagocytosed by alveolar MФ, at which point the bacilli can utilise this host immune cell as an immunoprivileged niche. Neighbouring immune cells are recruited to the infection site, whereby the granuloma is formed. This structure is initiated by MФ at the center, consolidated by the recruitment of other immune cells such as neutrophils, dendritic cells or B and T lymphocytes grouping together at the periphery of the granuloma (154–156). The protective or deleterious role of this structure has not yet been clearly defined. While some granulomas appear to control the infection and completely constrain the mycobacteria for many years, others overflow and eventually rupture, releasing the pathogen into the extracellular environment. These granulomas not only promote the spread and dissemination of the bacilli throughout the body and in the environment through the sputum but also generate cavities and irreversible lung lesions (157). Thus, the granuloma represents a major determinant for the disease outcome, formation of lesions and ultimately tissue restoration where MФ play a central role. Pro-inflammatory MФ are likely to promote granuloma formation and exerting a bactericidal activity *in vitro*, whereas anti-inflammatory MФ inhibit these effects (158–161). The role and polarization of MФ in granulomas in the presence of *M. tuberculosis* requires further attention, however studying the role of these structures remains complex due to the important cellular heterogeneity existing between individuals. In addition, murine models, although very useful for studying this pathology, incompletely reproduce the mechanisms associated with human granulomatous infiltrations, therefore limiting studies devoted to regeneration of the pulmonary epithelium in TB (162).

## SARS-CoV-2

SARS-CoV-2, the causative agent of Coronavirus Disease 2019 (COVID-19), is responsible for >180 million infections and 3.8 million deaths worldwide since its emergence in late 2019. Clinical manifestation is variable, ranging from asymptomatic or mild disease with moderate respiratory distress, to severe and life-threatening disease resulting in ARDS, acute lung injury and the need for mechanical ventilation, which in some cases results in death. Patients with moderate and severe COVID-19 have reported extended recovery periods and relapsing symptoms, but the underlying mechanisms remain unknown. Most current research has concentrated on understanding SARS-CoV-2 pathogenesis and developing effective vaccines and therapeutics to prevent or combat the disease. Unfortunately, there is still a limited understanding of the long-term effects following COVID-19 recovery.

Excessive inflammatory responses are a critical feature of severe COVID-19 and postulated to contribute to patient’s decline and death. The significant influx of inflammatory cells within alveoli leads to interstitial pneumonia, obstructing efficient gas exchange. Furthermore, ARDS is a known contributing factor to pulmonary fibrosis and is directly correlated with increased tissue resistance and substantial deterioration in the lung function. Recently, several representative animal models for SARS-CoV-2 pathogenesis have been identified, which recapitulate critical aspects of disease pathogenesis including extensive lung inflammation, severe lung damage and pulmonary fibrosis as well as significant pulmonary decline (163). This has been further demonstrated in COVID-19 mouse models, whereby significant changes to pulmonary function have been demonstrated to occur only during the latter stages of disease progression, coinciding with increased lung inflammatory infiltration and extensive interstitial inflammation (164). However, retrospective studies showed that AT2 epithelial cells were Ki67^+^, demonstrating that these cells were actively replicating following SARS-CoV-2 infection, suggesting that there is at least some degree of alveolar regeneration following COVID-19 resolution (165). Whether the extensive COVID-19 lung damage is able to regress over time, resulting in the restoration of the pulmonary lung function remains unknown.

## Conclusion

Understanding the processes associated with regeneration in mammals to improve regenerative capacities in humans, is one of the greatest challenges of the 21st century. Indeed, most degenerative diseases, accentuated with aging of the population, do not have therapies. Several major infectious diseases in humans, such as TB or SARS-CoV-2, are also responsible for tissue or lung damage, requiring regeneration. Restoration of tissues/organs through regenerative mechanisms, and not by repair, offers the functionality of the original structure to be regained. As a consequence, there is increasing interest in elucidating these mechanisms, which will be of high medical value. Numerous studies have already identified key mechanisms to understand the crucial role of the immune cells, such as MФ, in regeneration. However, the vast majority of these studies have been carried out under non-infectious conditions, which do not necessarily recapitulate the complexity of injuries encountered in humans. Therefore, the implementation of studies reproducing systemic and local infectious pathologies associated with injuries are particularly warranted. In the long term, it is anticipated that such studies may lead to the discovery of new regenerative therapies and/or improve the recovery state as seen with COVID-19 patients.

## Author Contributions

CB, NI, and FD propose the structure of the review. CB, MJ, CJ, LK, NI, and FD wrote the review. All authors contributed to the article and approved the submitted version.

## Conflict of Interest

The authors declare that the research was conducted in the absence of any commercial or financial relationships that could be construed as a potential conflict of interest.

## References

[1] Erickson JR Echeverri K. Learning From Regeneration Research Organisms: The Circuitous Road to Scar Free Wound Healing. Dev Biol (2018) 433:144–54. 10.1016/j.ydbio.2017.09.025 PMC5914521 29179946

[2] Iismaa SE Kaidonis X Nicks AM Bogush N Kikuchi K Naqvi N. Comparative Regenerative Mechanisms Across Different Mammalian Tissues. NPJ Regener Med (2018) 3:6. 10.1038/s41536-018-0044-5 PMC5824955 29507774

[3] Chan WY Lee KK Tam PP. Regenerative Capacity of Forelimb Buds After Amputation in Mouse Embryos at the Early-Organogenesis Stage. J Exp Xoology (1991) 260:74–83. 10.1002/jez.1402600110 1791423

[4] Lagrota-Candido J Canella I Pinheiro DF Santos-Silva LP Ferreira RS Guimaraes-Joca FJ. Characteristic Pattern of Skeletal Muscle Remodelling in Different Mouse Strains. Int J Exp Pathol (2010) 91:522–9. 10.1111/j.1365-2613.2010.00737.x PMC3010551 20804543

[5] Sciorati C Rigamonti E Manfredi AA Rovere-Querini P death C. Clearance and Immunity in the Skeletal Muscle. Cell Death Differ (2016) 23:927–37. 10.1038/cdd.2015.171 PMC4987728 26868912

[6] Hardy D Besnard A Latil M Jouvion G Briand D Thepenier C. Comparative Study of Injury Models for Studying Muscle Regeneration in Mice. PloS One (2016) 11:e0147198. 10.1371/journal.pone.0147198 26807982 PMC4726569

[7] Shen H Kreisel D Goldstein DR. Processes of Sterile Inflammation. J Immunol (2013) 191:2857–63. 10.4049/jimmunol.1301539 PMC3787118 24014880

[8] Matzinger P. Tolerance, Danger, and the Extended Family. Annu Rev Immunol (1994) 12:991–1045. 10.1146/annurev.iy.12.040194.005015 8011301

[9] Matzinger P. The Danger Model: A Renewed Sense of Self. Science (2002) 296:301–5. 10.1126/science.1071059 11951032

[10] Scaffidi P Misteli T Bianchi ME. Release of Chromatin Protein HMGB1 by Necrotic Cells Triggers Inflammation. Nature (2002) 418:191–5. 10.1038/nature00858 12110890

[11] Eigenbrod T Park JH Harder J Iwakura Y Nunez G. Cutting Edge: Critical Role for Mesothelial Cells in Necrosis-Induced Inflammation Through the Recognition of IL-1 Alpha Released From Dying Cells. J Immunol (2008) 181:8194–8. 10.4049/jimmunol.181.12.8194 PMC2762646 19050234

[12] Moussion C Ortega N Girard JP. The IL-1-Like Cytokine IL-33 Is Constitutively Expressed in the Nucleus of Endothelial Cells and Epithelial Cells *In Vivo*: A Novel ‘Alarmin’? PloS One (2008) 3:e3331. 10.1371/journal.pone.0003331 18836528 PMC2556082

[13] Gong T Liu L Jiang W Zhou R. DAMP-Sensing Receptors in Sterile Inflammation and Inflammatory Diseases. Nat Rev Immunol (2020) 20:95–112. 10.1038/s41577-019-0215-7 31558839

[14] Mogensen TH. Pathogen Recognition and Inflammatory Signaling in Innate Immune Defenses. Clin Microbiol Rev (2009) 22:240–73. 10.1128/CMR.00046-08 PMC2668232 19366914

[15] Silva-Gomes S Decout A Nigou J. Pathogen-Associated Molecular Patterns (PAMPs). In: Parnham M, editor. Encyclopedia of Inflammatory Diseases. Basel: Springer Basel (2015). p. 1–16. 10.1007/978-3-0348-0620-6_35-1

[16] Wang J. Neutrophils in Tissue Injury and Repair. Cell Tissue Res (2018) 371:531–9. 10.1007/s00441-017-2785-7 PMC5820392 29383445

[17] Mortaz E Alipoor SD Adcock IM Mumby S Koenderman L. Update on Neutrophil Function in Severe Inflammation. Front Immunol (2018) 9:2171. 10.3389/fimmu.2018.02171 30356867 PMC6190891

[18] Mantovani A Cassatella MA Costantini C Jaillon S. Neutrophils in the Activation and Regulation of Innate and Adaptive Immunity. Nat Rev Immunol (2011) 11:519–31. 10.1038/nri3024 21785456

[19] Leppkes M Schick M Hohberger B Mahajan A Knopf J Schett G. Updates on NET Formation in Health and Disease. Semin Arthritis Rheum (2019) 49:S43–8. 10.1016/j.semarthrit.2019.09.011 31779852

[20] Wang J Hossain M Thanabalasuriar A Gunzer M Meininger C Kubes P. Visualizing the Function and Fate of Neutrophils in Sterile Injury and Repair. Science (2017) 358:111–6. 10.1126/science.aam9690 28983053

[21] Wilkinson PC Borel JF Stecher-Levin VJ Sorkin E. Macrophage and Neutrophil Specific Chemotactic Factors in Serum. Nature (1969) 222:244–7. 10.1038/222244a0 5778389

[22] Chertov O Ueda H Xu LL Tani K Murphy WJ Wang JM. Identification of Human Neutrophil-Derived Cathepsin G and Azurocidin/CAP37 as Chemoattractants for Mononuclear Cells and Neutrophils. J Exp Med (1997) 186:739–47. 10.1084/jem.186.5.739 PMC2199011 9271589

[23] Soehnlein O Zernecke A Eriksson EE Rothfuchs AG Pham CT Herwald H. Neutrophil Secretion Products Pave the Way for Inflammatory Monocytes. Blood (2008) 112:1461–71. 10.1182/blood-2008-02-139634 PMC3400540 18490516

[24] Moore BB Moore TA Toews GB. Role of T- and B-Lymphocytes in Pulmonary Host Defences. Eur Respir J (2001) 18:846–56. 10.1183/09031936.01.00229001 11757636

[25] Fox S Leitch AE Duffin R Haslett C Rossi AG. Neutrophil Apoptosis: Relevance to the Innate Immune Response and Inflammatory Disease. J Innate Immun (2010) 2:216–27. 10.1159/000284367 PMC2956014 20375550

[26] Bellingan GJ Laurent GJ. Fate of Macrophages Once Having Ingested Apoptotic Cells: Lymphatic Clearance or in Situ Apoptosis? In: Rossi AG Sawatzky DA, editors. The Resolution of Inflammation. Basel: Birkhäuser Basel (2008). p. 75–91. 10.1007/978-3-7643-7506-5_5

[27] Cicchese JM Evans S Hult C Joslyn LR Wessler T Millar JA. Dynamic Balance of Pro- and Anti-Inflammatory Signals Controls Disease and Limits Pathology. Immunol Rev (2018) 285:147–67. 10.1111/imr.12671 PMC6292442 30129209

[28] Karin M Clevers H. Reparative Inflammation Takes Charge of Tissue Regeneration. Nature (2016) 529:307–15. 10.1038/nature17039 PMC5228603 26791721

[29] Epelman S Lavine KJ Randolph GJ. Origin and Functions of Tissue Macrophages. Immunity (2014) 41:21–35. 10.1016/j.immuni.2014.06.013 25035951 PMC4470379

[30] Lavin Y Winter D Blecher-Gonen R David E Keren-Shaul H Merad M. Tissue-Resident Macrophage Enhancer Landscapes Are Shaped by the Local Microenvironment. Cell (2014) 159:1312–26. 10.1016/j.cell.2014.11.018 PMC4437213 25480296

[31] Kierdorf K Prinz M Geissmann F Gomez Perdiguero E. Development and Function of Tissue Resident Macrophages in Mice. Semin Immunol (2015) 27:369–78. 10.1016/j.smim.2016.03.017 PMC4948121 27036090

[32] Ginhoux F Guilliams M. Tissue-Resident Macrophage Ontogeny and Homeostasis. Immunity (2016) 44:439–49. 10.1016/j.immuni.2016.02.024 26982352

[33] Gordon S Martinez-Pomares L. Physiological Roles of Macrophages. Pflugers Arch (2017) 469:365–74. 10.1007/s00424-017-1945-7 PMC5362657 28185068

[34] Davies LC Jenkins SJ Allen JE Taylor PR. Tissue-Resident Macrophages. Nat Immunol (2013) 14:986–95. 10.1038/ni.2705 PMC4045180 24048120

[35] Davies LC Taylor PR. Tissue-Resident Macrophages: Then and Now. Immunology (2015) 144:541–8. 10.1111/imm.12451 PMC4368161 25684236

[36] Beck-Schimmer B Schwendener R Pasch T Reyes L Booy C Schimmer RC. Alveolar Macrophages Regulate Neutrophil Recruitment in Endotoxin-Induced Lung Injury. Respir Res (2005) 6:61. 10.1186/1465-9921-6-61 15972102 PMC1188075

[37] De Filippo K Henderson RB Laschinger M Hogg N Neutrophil chemokines KC. And Macrophage-Inflammatory Protein-2 Are Newly Synthesized by Tissue Macrophages Using Distinct TLR Signaling Pathways. J Immunol (2008) 180:4308–15. 10.4049/jimmunol.180.6.4308 18322244

[38] De Filippo K Dudeck A Hasenberg M Nye E van Rooijen N Hartmann K. Mast Cell and Macrophage Chemokines CXCL1/CXCL2 Control the Early Stage of Neutrophil Recruitment During Tissue Inflammation. Blood (2013) 121:4930–7. 10.1182/blood-2013-02-486217 23645836

[39] Prame Kumar K Nicholls AJ Wong CHY. Partners in Crime: Neutrophils and Monocytes/Macrophages in Inflammation and Disease. Cell Tissue Res (2018) 371:551–65. 10.1007/s00441-017-2753-2 PMC5820413 29387942

[40] Porcheray F Viaud S Rimaniol AC Leone C Samah B Dereuddre-Bosquet N. Macrophage Activation Switching: An Asset for the Resolution of Inflammation. Clin Exp Immunol (2005) 142:481–9. 10.1111/j.1365-2249.2005.02934.x PMC1809537 16297160

[41] Xue J Schmidt SV Sander J Draffehn A Krebs W Quester I. Transcriptome-Based Network Analysis Reveals a Spectrum Model of Human Macrophage Activation. Immunity (2014) 40:274–88. 10.1016/j.immuni.2014.01.006 PMC3991396 24530056

[42] Watanabe S Alexander M Misharin AV Budinger GRS. The Role of Macrophages in the Resolution of Inflammation. J Clin Invest (2019) 129:2619–28. 10.1172/JCI124615 PMC6597225 31107246

[43] Arnold L Henry A Poron F Baba-Amer Y van Rooijen N Plonquet A. Inflammatory Monocytes Recruited After Skeletal Muscle Injury Switch Into Antiinflammatory Macrophages to Support Myogenesis. J Exp Med (2007) 204:1057–69. 10.1084/jem.20070075 PMC2118577 17485518

[44] Marwick JA Mills R Kay O Michail K Stephen J Rossi AG. Neutrophils Induce Macrophage Anti-Inflammatory Reprogramming by Suppressing NF-KappaB Activation. Cell Death Dis (2018) 9:665. 10.1038/s41419-018-0710-y 29867198 PMC5986789

[45] Nahrendorf M Swirski FK Aikawa E Stangenberg L Wurdinger T Figueiredo JL. The Healing Myocardium Sequentially Mobilizes Two Monocyte Subsets With Divergent and Complementary Functions. J Exp Med (2007) 204:3037–47. 10.1084/jem.20070885 PMC2118517 18025128

[46] Lech M Anders HJ. Macrophages and Fibrosis: How Resident and Infiltrating Mononuclear Phagocytes Orchestrate All Phases of Tissue Injury and Repair. Biochim Biophys Acta (2013) 1832:989–97. 10.1016/j.bbadis.2012.12.001 23246690

[47] Atri C Guerfali FZ Laouini D. Role of Human Macrophage Polarization in Inflammation During Infectious Diseases. Int J Mol Sci (2018) 19(6):1801–15. 10.3390/ijms19061801 PMC6032107 29921749

[48] Robson MC. Wound Infection. A Failure of Wound Healing Caused by an Imbalance of Bacteria. Surg Clin North Am (1997) 77:637–50. 10.1016/S0039-6109(05)70572-7 9194884

[49] Bankey P Fiegel V Singh R Knighton D Cerra F. Hypoxia and Endotoxin Induce Macrophage-Mediated Suppression of Fibroblast Proliferation. J Trauma (1989) 29:972–9; discussion 979-80. 10.1097/00005373-198907000-00011 2664203

[50] Kwee BJ Mooney DJ. Biomaterials for Skeletal Muscle Tissue Engineering. Curr Opin Biotechnol (2017) 47:16–22. 10.1016/j.copbio.2017.05.003 28575733 PMC5617779

[51] Tedesco FS Dellavalle A Diaz-Manera J Messina G Cossu G. Repairing Skeletal Muscle: Regenerative Potential of Skeletal Muscle Stem Cells. J Clin Invest (2010) 120:11–9. 10.1172/JCI40373 PMC2798695 20051632

[52] Buckingham M Relaix F. PAX3 and PAX7 as Upstream Regulators of Myogenesis. Semin Cell Dev Biol (2015) 44:115–25. 10.1016/j.semcdb.2015.09.017 26424495

[53] Relaix F Zammit PS. Satellite Cells Are Essential for Skeletal Muscle Regeneration: The Cell on the Edge Returns Centre Stage. Development (2012) 139:2845–56. 10.1242/dev.069088 22833472

[54] Abmayr SM Pavlath GK. Myoblast Fusion: Lessons From Flies and Mice. Development (2012) 139:641–56. 10.1242/dev.068353 PMC3265056 22274696

[55] Chal J Pourquie O. Making Muscle: Skeletal Myogenesis *In Vivo* and *In Vitro* . Development (2017) 144:2104–22. 10.1242/dev.151035 28634270

[56] Chal J Oginuma M Al Tanoury Z Gobert B Sumara O Hick A. Differentiation of Pluripotent Stem Cells to Muscle Fiber to Model Duchenne Muscular Dystrophy. Nat Biotechnol (2015) 33:962–9. 10.1038/nbt.3297 26237517

[57] Honda H Kimura H Rostami A. Demonstration and Phenotypic Characterization of Resident Macrophages in Rat Skeletal Muscle. Immunology (1990) 70:272–7. PMC1384205 2197218

[58] Honda H Kimura H Silvers WK Rostami A. Perivascular Location and Phenotypic Heterogeneity of Microglial Cells in the Rat Brain. J Neuroimmunol (1990) 29:183–91. 10.1016/0165-5728(90)90161-F 2104520

[59] Przybyla B Gurley C Harvey JF Bearden E Kortebein P Evans WJ. Aging Alters Macrophage Properties in Human Skeletal Muscle Both at Rest and in Response to Acute Resistance Exercise. Exp Gerontol (2006) 41:320–7. 10.1016/j.exger.2005.12.007 16457979

[60] Theret M Mounier R Rossi F. The Origins and Non-Canonical Functions of Macrophages in Development and Regeneration. Dev (2019) 146(9):1–14. 10.1242/dev.156000 31048317

[61] Zuniga-Pereira AM Santamaria C Gutierrez JM Alape-Giron A Flores-Diaz M. Deficient Skeletal Muscle Regeneration After Injury Induced by a Clostridium Perfringens Strain Associated With Gas Gangrene. Infect Immun (2019) 87(8):e00200–19. 10.1128/IAI.00200-19 PMC6652765 31138614

[62] Summan M Warren GL Mercer RR Chapman R Hulderman T Van Rooijen N. Macrophages and Skeletal Muscle Regeneration: A Clodronate-Containing Liposome Depletion Study. Am J Physiol Regulatory Integr Comp Physiol (2006) 290:R1488–95. 10.1152/ajpregu.00465.2005 16424086

[63] Segawa M Fukada S Yamamoto Y Yahagi H Kanematsu M Sato M. Suppression of Macrophage Functions Impairs Skeletal Muscle Regeneration With Severe Fibrosis. Exp Cell Res (2008) 314:3232–44. 10.1016/j.yexcr.2008.08.008 18775697

[64] Melton DW Roberts AC Wang H Sarwar Z Wetzel MD Wells JT. Absence of CCR2 Results in an Inflammaging Environment in Young Mice With Age-Independent Impairments in Muscle Regeneration. J Leukocyte Biol (2016) 100:1011–25. 10.1189/jlb.3MA0316-104R PMC5069084 27531927

[65] Chazaud B. Inflammation and Skeletal Muscle Regeneration: Leave It to the Macrophages! Trends Immunol (2020) 41:481–92. 10.1016/j.it.2020.04.006 32362490

[66] Martinez CO McHale MJ Wells JT Ochoa O Michalek JE McManus LM. Regulation of Skeletal Muscle Regeneration by CCR2-Activating Chemokines Is Directly Related to Macrophage Recruitment. Am J Physiol Regulatory Integr Comp Physiol (2010) 299:R832–42. 10.1152/ajpregu.00797.2009 PMC2944434 20631294

[67] Ochoa O Sun D Reyes-Reyna SM Waite LL Michalek JE McManus LM. Delayed Angiogenesis and VEGF Production in CCR2-/- Mice During Impaired Skeletal Muscle Regeneration. Am J Physiol Regulatory Integr Comp Physiol (2007) 293:R651–61. 10.1152/ajpregu.00069.2007 17522124

[68] McLennan IS. Resident Macrophages (ED2- and ED3-Positive) Do Not Phagocytose Degenerating Rat Skeletal Muscle Fibres. Cell Tissue Res (1993) 272:193–6. 10.1007/BF00323586 8481952

[69] Brigitte M Schilte C Plonquet A Baba-Amer Y Henri A Charlier C. Muscle Resident Macrophages Control the Immune Cell Reaction in a Mouse Model of Notexin-Induced Myoinjury. Arthritis Rheumatism (2010) 62:268–79. 10.1002/art.27183 20039420

[70] Li YP. TNF-Alpha Is a Mitogen in Skeletal Muscle. Am J Physiol Cell Physiol (2003) 285:C370–6. 10.1152/ajpcell.00453.2002 12711593

[71] Szalay K Razga Z Duda E. TNF Inhibits Myogenesis and Downregulates the Expression of Myogenic Regulatory Factors myoD and Myogenin. Eur J Cell Biol (1997) 74:391–8. 9438136

[72] Serrano AL Baeza-Raja B Perdiguero E Jardi M Munoz-Canoves P. Interleukin-6 Is an Essential Regulator of Satellite Cell-Mediated Skeletal Muscle Hypertrophy. Cell Metab (2008) 7:33–44. 10.1016/j.cmet.2007.11.011 18177723

[73] Hara M Yuasa S Shimoji K Onizuka T Hayashiji N Ohno Y. G-CSF Influences Mouse Skeletal Muscle Development and Regeneration by Stimulating Myoblast Proliferation. J Exp Med (2011) 208:715–27. 10.1084/jem.20101059 PMC3135344 21422169

[74] Otis JS Niccoli S Hawdon N Sarvas JL Frye MA Chicco AJ. Pro-Inflammatory Mediation of Myoblast Proliferation. PloS One (2014) 9:e92363. 10.1371/journal.pone.0092363 24647690 PMC3960233

[75] Pelosi L Giacinti C Nardis C Borsellino G Rizzuto E Nicoletti C. Local Expression of IGF-1 Accelerates Muscle Regeneration by Rapidly Modulating Inflammatory Cytokines and Chemokines. FASEB J: Off Publ Fed Am Soc Exp Biol (2007) 21:1393–402. 10.1096/fj.06-7690com 17264161

[76] Lu H Huang D Saederup N Charo IF Ransohoff RM Zhou L. Macrophages Recruited *via* CCR2 Produce Insulin-Like Growth Factor-1 to Repair Acute Skeletal Muscle Injury. FASEB J: Off Publ Fed Am Soc Exp Biol (2011) 25:358–69. 10.1096/fj.10-171579 PMC3005436 20889618

[77] Novak ML Weinheimer-Haus EM Koh TJ. Macrophage Activation and Skeletal Muscle Healing Following Traumatic Injury. J Pathol (2014) 232:344–55. 10.1002/path.4301 PMC4019602 24255005

[78] Mounier R Theret M Arnold L Cuvellier S Bultot L Goransson O. AMPKalpha1 Regulates Macrophage Skewing at the Time of Resolution of Inflammation During Skeletal Muscle Regeneration. Cell Metab (2013) 18:251–64. 10.1016/j.cmet.2013.06.017 23931756

[79] Ruffell D Mourkioti F Gambardella A Kirstetter P Lopez RG Rosenthal N. A CREB-C/EBPbeta Cascade Induces M2 Macrophage-Specific Gene Expression and Promotes Muscle Injury Repair. Proc Natl Acad Sci USA (2009) 106:17475–80. 10.1073/pnas.0908641106 PMC2762675 19805133

[80] Panduro M Benoist C Mathis D. Treg Cells Limit IFN-Gamma Production to Control Macrophage Accrual and Phenotype During Skeletal Muscle Regeneration. Proc Natl Acad Sci USA (2018) 115:E2585–93. 10.1073/pnas.1800618115 PMC5856564 29476012

[81] Joe AW Yi L Natarajan A Le Grand F So L Wang J. Muscle Injury Activates Resident Fibro/Adipogenic Progenitors That Facilitate Myogenesis. Nat Cell Biol (2010) 12:153–63. 10.1038/ncb2015 PMC4580288 20081841

[82] Lemos DR Babaeijandaghi F Low M Chang CK Lee ST Fiore D. Nilotinib Reduces Muscle Fibrosis in Chronic Muscle Injury by Promoting TNF-Mediated Apoptosis of Fibro/Adipogenic Progenitors. Nat Med (2015) 21:786–94. 10.1038/nm.3869 26053624

[83] Howard EE Pasiakos SM Blesso CN Fussell MA Rodriguez NR. Divergent Roles of Inflammation in Skeletal Muscle Recovery From Injury. Front Physiol (2020) 11:87. 10.3389/fphys.2020.00087 32116792 PMC7031348

[84] Buboltz JB Murphy-Lavoie HM. Gas Gangrene. Treasure Island (FL: StatPearls (2020). 30725715

[85] Stevens DL Bryant AE. Necrotizing Soft-Tissue Infections. N Engl J Med (2017) 377:2253–65. 10.1056/NEJMra1600673 29211672

[86] Jin RM Blair SJ Warunek J Heffner RR Blader IJ Wohlfert EA. Regulatory T Cells Promote Myositis and Muscle Damage in *Toxoplasma gondii* Infection. J Immunol (2017) 198:352–62. 10.4049/jimmunol.1600914 PMC5173414 27895180

[87] Hatter JA Kouche YM Melchor SJ Ng K Bouley DM Boothroyd JC. *Toxoplasma gondii* Infection Triggers Chronic Cachexia and Sustained Commensal Dysbiosis in Mice. PloS One (2018) 13:e0204895. 10.1371/journal.pone.0204895 30379866 PMC6209157

[88] Jin RM Warunek J Wohlfert EA. Chronic Infection Stunts Macrophage Heterogeneity and Disrupts Immune-Mediated Myogenesis. JCI Insight (2018) 3(18):1–22. 10.1172/jci.insight.121549 PMC6237226 30232283

[89] Hogan BL Barkauskas CE Chapman HA Epstein JA Jain R Hsia CC. Repair and Regeneration of the Respiratory System: Complexity, Plasticity, and Mechanisms of Lung Stem Cell Function. Cell Stem Cell (2014) 15:123–38. 10.1016/j.stem.2014.07.012 PMC4212493 25105578

[90] Morrisey EE Hogan BL. Preparing for the First Breath: Genetic and Cellular Mechanisms in Lung Development. Dev Cell (2010) 18:8–23. 10.1016/j.devcel.2009.12.010 20152174 PMC3736813

[91] Nikolic MZ Sun D Rawlins EL. Human Lung Development: Recent Progress and New Challenges. Development (2018) 145(16):1–14. 10.1242/dev.163485 PMC6124546 30111617

[92] Hegab AE Nickerson DW Ha VL Darmawan DO Gomperts BN. Repair and Regeneration of Tracheal Surface Epithelium and Submucosal Glands in a Mouse Model of Hypoxic-Ischemic Injury. Respirology (2012) 17:1101–13. 10.1111/j.1440-1843.2012.02204.x PMC3723929 22617027

[93] Hong KU Reynolds SD Watkins S Fuchs E Stripp BR. *In Vivo* Differentiation Potential of Tracheal Basal Cells: Evidence for Multipotent and Unipotent Subpopulations. Am J Physiol Lung Cell Mol Physiol (2004) 286:L643–9. 10.1152/ajplung.00155.2003 12871857

[94] Rock JR Gao X Xue Y Randell SH Kong YY Hogan BL. Notch-Dependent Differentiation of Adult Airway Basal Stem Cells. Cell Stem Cell (2011) 8:639–48. 10.1016/j.stem.2011.04.003 PMC3778678 21624809

[95] Rock JR Onaitis MW Rawlins EL Lu Y Clark CP Xue Y. Basal Cells as Stem Cells of the Mouse Trachea and Human Airway Epithelium. Proc Natl Acad Sci USA (2009) 106:12771–5. 10.1073/pnas.0906850106 PMC2714281 19625615

[96] Barkauskas CE Cronce MJ Rackley CR Bowie EJ Keene DR Stripp BR. Type 2 Alveolar Cells Are Stem Cells in Adult Lung. J Clin Invest (2013) 123:3025–36. 10.1172/JCI68782 PMC3696553 23921127

[97] Rawlins EL Okubo T Xue Y Brass DM Auten RL Hasegawa H. The Role of Scgb1a1+ Clara Cells in the Long-Term Maintenance and Repair of Lung Airway, But Not Alveolar, Epithelium. Cell Stem Cell (2009) 4:525–34. 10.1016/j.stem.2009.04.002 PMC2730729 19497281

[98] Wansleeben C Barkauskas CE Rock JR Hogan BL. Stem Cells of the Adult Lung: Their Development and Role in Homeostasis, Regeneration, and Disease. Wiley Interdiscip Rev Dev Biol (2013) 2:131–48. 10.1002/wdev.58 23799633

[99] Hogan BLM. The Alveolar Stem Cell Niche of the Mammalian Lung. Singapore: Springer Singapore (2020). p. 7–12. 10.1007/978-981-15-1185-1_2

[100] Morimoto M. Lung Development and Notch Signaling. Singapore: Springer Singapore (2020). p. 13–23. 10.1007/978-981-15-1185-1_3

[101] Kotton DN Morrisey EE. Lung Regeneration: Mechanisms, Applications and Emerging Stem Cell Populations. Nat Med (2014) 20:822–32. 10.1038/nm.3642 PMC4229034 25100528

[102] Matute-Bello G Frevert CW Martin TR. Animal Models of Acute Lung Injury. Am J Physiol Lung Cell Mol Physiol (2008) 295:L379–99. 10.1152/ajplung.00010.2008 PMC2536793 18621912

[103] Bastarache JA Blackwell TS. Development of Animal Models for the Acute Respiratory Distress Syndrome. Dis Model Mech (2009) 2:218–23. 10.1242/dmm.001677 PMC2675821 19407329

[104] Matute-Bello G Downey G Moore BB Groshong SD Matthay MA Slutsky AS. An Official American Thoracic Society Workshop Report: Features and Measurements of Experimental Acute Lung Injury in Animals. Am J Respir Cell Mol Biol (2011) 44:725–38. 10.1165/rcmb.2009-0210ST PMC7328339 21531958

[105] Dagher R Copenhaver AM Besnard V Berlin A Hamidi F Maret M. IL-33-ST2 Axis Regulates Myeloid Cell Differentiation and Activation Enabling Effective Club Cell Regeneration. Nat Commun (2020) 11:4786. 10.1038/s41467-020-18466-w 32963227 PMC7508874

[106] Atif SM Gibbings SL Jakubzick CV. Isolation and Identification of Interstitial Macrophages From the Lungs Using Different Digestion Enzymes and Staining Strategies. Methods Mol Biol (2018) 1784:69–76. 10.1007/978-1-4939-7837-3_6 29761388 PMC6233879

[107] Weaver TE Whitsett JA. Function and Regulation of Expression of Pulmonary Surfactant-Associated Proteins. Biochem J 273(Pt (1991) 2):249–64. 10.1042/bj2730249 PMC1149839 1991023

[108] Guilliams M De Kleer I Henri S Post S Vanhoutte L De Prijck S. Alveolar Macrophages Develop From Fetal Monocytes That Differentiate Into Long-Lived Cells in the First Week of Life *via* GM-CSF. J Exp Med (2013) 210:1977–92. 10.1084/jem.20131199 PMC3782041 24043763

[109] Schneider C Nobs SP Kurrer M Rehrauer H Thiele C Kopf M. Induction of the Nuclear Receptor PPAR-Gamma by the Cytokine GM-CSF Is Critical for the Differentiation of Fetal Monocytes Into Alveolar Macrophages. Nat Immunol (2014) 15:1026–37. 10.1038/ni.3005 25263125

[110] Yu X Buttgereit A Lelios I Utz SG Cansever D Becher B. The Cytokine TGF-Beta Promotes the Development and Homeostasis of Alveolar Macrophages. Immunity (2017) 47:903–12.e4. 10.1016/j.immuni.2017.10.007 29126797

[111] Gibbings SL Thomas SM Atif SM McCubbrey AL Desch AN Danhorn T. Three Unique Interstitial Macrophages in the Murine Lung at Steady State. Am J Respir Cell Mol Biol (2017) 57:66–76. 10.1165/rcmb.2016-0361OC 28257233 PMC5516280

[112] Chakarov S Lim HY Tan L Lim SY See P Lum J. Two Distinct Interstitial Macrophage Populations Coexist Across Tissues in Specific Subtissular Niches. Science (2019) 363(6432):eaau0964. 10.1126/science.aau0964 30872492

[113] Tan SY Krasnow MA. Developmental Origin of Lung Macrophage Diversity. Development (2016) 143:1318–27. 10.1242/dev.129122 PMC4852511 26952982

[114] Schyns J Bureau F Marichal T. Lung Interstitial Macrophages: Past, Present, and Future. J Immunol Res (2018) 2018:5160794. 10.1155/2018/5160794 29854841 PMC5952507

[115] Hashimoto D Chow A Noizat C Teo P Beasley MB Leboeuf M. Tissue-Resident Macrophages Self-Maintain Locally Throughout Adult Life With Minimal Contribution From Circulating Monocytes. Immunity (2013) 38:792–804. 10.1016/j.immuni.2013.04.004 23601688 PMC3853406

[116] Janssen WJ Barthel L Muldrow A Oberley-Deegan RE Kearns MT Jakubzick C. Fas Determines Differential Fates of Resident and Recruited Macrophages During Resolution of Acute Lung Injury. Am J Respir Crit Care Med (2011) 184:547–60. 10.1164/rccm.201011-1891OC PMC3175550 21471090

[117] Maus UA Janzen S Wall G Srivastava M Blackwell TS Christman JW. Resident Alveolar Macrophages Are Replaced by Recruited Monocytes in Response to Endotoxin-Induced Lung Inflammation. Am J Respir Cell Mol Biol (2006) 35:227–35. 10.1165/rcmb.2005-0241OC 16543608

[118] Misharin AV Morales-Nebreda L Reyfman PA Cuda CM Walter JM McQuattie-Pimentel AC. Monocyte-Derived Alveolar Macrophages Drive Lung Fibrosis and Persist in the Lung Over the Life Span. J Exp Med (2017) 214:2387–404. 10.1084/jem.20162152 PMC5551573 28694385

[119] Reiss LK Kowallik A Uhlig S. Recurrent Recruitment Manoeuvres Improve Lung Mechanics and Minimize Lung Injury During Mechanical Ventilation of Healthy Mice. PloS One (2011) 6:e24527. 10.1371/journal.pone.0024527 21935418 PMC3174196

[120] Amigoni M Bellani G Scanziani M Masson S Bertoli E Radaelli E. Lung Injury and Recovery in a Murine Model of Unilateral Acid Aspiration: Functional, Biochemical, and Morphologic Characterization. Anesthesiology (2008) 108:1037–46. 10.1097/ALN.0b013e318173f64f 18497604

[121] Border JR Hopkinson BR Schenk WG Jr. Mechanisms of Pulmonary Trauma. An Experimental Study. J Trauma (1968) 8:47–62. 10.1097/00005373-196801000-00006 5638141

[122] Raghavendran K Davidson BA Helinski JD Marschke CJ Manderscheid P Woytash JA. A Rat Model for Isolated Bilateral Lung Contusion From Blunt Chest Trauma. Anesth Analg (2005) 101:1482–9. 10.1213/01.ANE.0000180201.25746.1F 16244015

[123] Fitschen-Oestern S Lippross S Klueter T Weuster M Varoga D Tohidnezhad M. A New Multiple Trauma Model of the Mouse. BMC Musculoskelet Disord (2017) 18:468. 10.1186/s12891-017-1813-9 29157219 PMC5697084

[124] Izbicki G Segel MJ Christensen TG Conner MW Breuer R. Time Course of Bleomycin-Induced Lung Fibrosis. Int J Exp Pathol (2002) 83:111–9. 10.1046/j.1365-2613.2002.00220.x PMC2517673 12383190

[125] Sun Y Tian Y Prabha M Liu D Chen S Zhang R. Effects of Sulfur Dioxide on Hypoxic Pulmonary Vascular Structural Remodeling. Lab Invest (2010) 90:68–82. 10.1038/labinvest.2009.102 19823174

[126] Martin JG Campbell HR Iijima H Gautrin D Malo JL Eidelman DH. Chlorine-Induced Injury to the Airways in Mice. Am J Respir Crit Care Med (2003) 168:568–74. 10.1164/rccm.200201-021OC 12724121

[127] Escolar JD Martinez MN Rodriguez FJ Gonzalo C Escolar MA Roche PA. Emphysema as a Result of Involuntary Exposure to Tobacco Smoke: Morphometrical Study of the Rat. Exp Lung Res (1995) 21:255–73. 10.3109/01902149509068831 7774528

[128] Cavarra E Bartalesi B Lucattelli M Fineschi S Lunghi B Gambelli F. Effects of Cigarette Smoke in Mice With Different Levels of Alpha(1)-Proteinase Inhibitor and Sensitivity to Oxidants. Am J Respir Crit Care Med (2001) 164:886–90. 10.1164/ajrccm.164.5.2010032 11549550

[129] Imanaka H Shimaoka M Matsuura N Nishimura M Ohta N Kiyono H. Ventilator-Induced Lung Injury Is Associated With Neutrophil Infiltration, Macrophage Activation, and TGF-β1 mRNA Upregulation in Rat Lungs. Anesthesia Analgesia (2001) 92:428–36. 10.1097/00000539-200102000-00029 11159246

[130] Frank JA Wray CM McAuley DF Schwendener R Matthay MA. Alveolar Macrophages Contribute to Alveolar Barrier Dysfunction in Ventilator-Induced Lung Injury. Am J Physiol Lung Cell Mol Physiol (2006) 291:L1191–8. 10.1152/ajplung.00055.2006 16877636

[131] Mohning MP Thomas SM Barthel L Mould KJ McCubbrey AL Frasch SC. Phagocytosis of Microparticles by Alveolar Macrophages During Acute Lung Injury Requires MerTK. Am J Physiol Lung Cell Mol Physiol (2018) 314:L69–82. 10.1152/ajplung.00058.2017 PMC6335009 28935638

[132] Melloni B Lesur O Bouhadiba T Cantin A Martel M Begin R. Effect of Exposure to Silica on Human Alveolar Macrophages in Supporting Growth Activity in Type II Epithelial Cells. Thorax (1996) 51:781–6. 10.1136/thx.51.8.781 PMC472535 8795664

[133] Hung LY Sen D Oniskey TK Katzen J Cohen NA Vaughan AE. Macrophages Promote Epithelial Proliferation Following Infectious and Non-Infectious Lung Injury Through a Trefoil Factor 2-Dependent Mechanism. Mucosal Immunol (2019) 12:64–76. 10.1038/s41385-018-0096-2 30337651 PMC6301101

[134] Gibbons MA MacKinnon AC Ramachandran P Dhaliwal K Duffin R Phythian-Adams AT. Ly6Chi Monocytes Direct Alternatively Activated Profibrotic Macrophage Regulation of Lung Fibrosis. Am J Respir Crit Care Med (2011) 184:569–81. 10.1164/rccm.201010-1719OC 21680953

[135] Lechner AJ Driver IH Lee J Conroy CM Nagle A Locksley RM. Recruited Monocytes and Type 2 Immunity Promote Lung Regeneration Following Pneumonectomy. Cell Stem Cell (2017) 21:120–34.e7. 10.1016/j.stem.2017.03.024 28506464 PMC5501755

[136] Allard B Panariti A Pernet E Downey J Ano S Dembele M. Tolerogenic Signaling of Alveolar Macrophages Induces Lung Adaptation to Oxidative Injury. J Allergy Clin Immunol (2019) 144:945–61.e9. 10.1016/j.jaci.2019.07.015 31356919

[137] Laskin DL Malaviya R Laskin JD. Role of Macrophages in Acute Lung Injury and Chronic Fibrosis Induced by Pulmonary Toxicants. Toxicol Sci (2019) 168:287–301. 10.1093/toxsci/kfy309 30590802 PMC6432864

[138] Redente EF Keith RC Janssen W Henson PM Ortiz LA Downey GP. Tumor Necrosis Factor-Alpha Accelerates the Resolution of Established Pulmonary Fibrosis in Mice by Targeting Profibrotic Lung Macrophages. Am J Respir Cell Mol Biol (2014) 50:825–37. 10.1165/rcmb.2013-0386OC PMC4068926 24325577

[139] Kumar PA Hu Y Yamamoto Y Hoe NB Wei TS Mu D. Distal Airway Stem Cells Yield Alveoli *In Vitro* and During Lung Regeneration Following H1N1 Influenza Infection. Cell (2011) 147:525–38. 10.1016/j.cell.2011.10.001 PMC4040224 22036562

[140] Shao H Qin Z Geng B Wu J Zhang L Zhang Q. Impaired Lung Regeneration After SARS-CoV-2 Infection. Cell Proliferation (2020) 53:e12927. 10.1111/cpr.12927 33078459 PMC7645888

[141] Pociask DA Scheller EV Mandalapu S McHugh KJ Enelow RI Fattman CL. IL-22 Is Essential for Lung Epithelial Repair Following Influenza Infection. Am J Pathol (2013) 182:1286–96. 10.1016/j.ajpath.2012.12.007 PMC3620404 23490254

[142] Mineo G Ciccarese F Modolon C Landini MP Valentino M Zompatori M. Post-ARDS Pulmonary Fibrosis in Patients With H1N1 Pneumonia: Role of Follow-Up CT. Radiol Med (2012) 117:185–200. 10.1007/s11547-011-0740-3 22020433 PMC7102178

[143] Pociask DA Robinson KM Chen K McHugh KJ Clay ME Huang GT. Epigenetic and Transcriptomic Regulation of Lung Repair During Recovery From Influenza Infection. Am J Pathol (2017) 187:851–63. 10.1016/j.ajpath.2016.12.012 PMC5397680 28193481

[144] Kim HM Lee YW Lee KJ Kim HS Cho SW van Rooijen N. Alveolar Macrophages Are Indispensable for Controlling Influenza Viruses in Lungs of Pigs. J Virol (2008) 82:4265–74. 10.1128/JVI.02602-07 PMC2293066 18287245

[145] Herold S Becker C Ridge KM Budinger GR. Influenza Virus-Induced Lung Injury: Pathogenesis and Implications for Treatment. Eur Respir J (2015) 45:1463–78. 10.1183/09031936.00186214 25792631

[146] Tumpey TM Garcia-Sastre A Taubenberger JK Palese P Swayne DE Pantin-Jackwood MJ. Pathogenicity of Influenza Viruses With Genes From the 1918 Pandemic Virus: Functional Roles of Alveolar Macrophages and Neutrophils in Limiting Virus Replication and Mortality in Mice. J Virol (2005) 79:14933–44. 10.1128/JVI.79.23.14933-14944.2005 PMC1287592 16282492

[147] Divangahi M King IL Pernet E. Alveolar Macrophages and Type I IFN in Airway Homeostasis and Immunity. Trends Immunol (2015) 36:307–14. 10.1016/j.it.2015.03.005 25843635

[148] Cakarova L Marsh LM Wilhelm J Mayer K Grimminger F Seeger W. Macrophage Tumor Necrosis Factor-Alpha Induces Epithelial Expression of Granulocyte-Macrophage Colony-Stimulating Factor: Impact on Alveolar Epithelial Repair. Am J Respir Crit Care Med (2009) 180:521–32. 10.1164/rccm.200812-1837OC 19590023

[149] Unkel B Hoegner K Clausen BE Lewe-Schlosser P Bodner J Gattenloehner S. Alveolar Epithelial Cells Orchestrate DC Function in Murine Viral Pneumonia. J Clin Invest (2012) 122:3652–64. 10.1172/JCI62139 PMC3461909 22996662

[150] Kulikauskaite J Wack A. Teaching Old Dogs New Tricks? The Plasticity of Lung Alveolar Macrophage Subsets. Trends Immunol (2020) 41:864–77. 10.1016/j.it.2020.08.008 PMC7472979 32896485

[151] Aegerter H Kulikauskaite J Crotta S Patel H Kelly G Hessel EM. Influenza-Induced Monocyte-Derived Alveolar Macrophages Confer Prolonged Antibacterial Protection. Nat Immunol (2020) 21:145–57. 10.1038/s41590-019-0568-x PMC6983324 31932810

[152] Ravimohan S Kornfeld H Weissman D Bisson GP. Tuberculosis and Lung Damage: From Epidemiology to Pathophysiology. Eur Respir Rev (2018) 27(170077):1–20. 10.1183/16000617.0077-2017 PMC6019552 29491034

[153] Bobrowitz ID Rodescu D Marcus H Abeles H. The Destroyed Tuberculous Lung. Scand J Respir Dis (1974) 55:82–8. 4854707

[154] Dannenberg AM Jr. Immunopathogenesis of Pulmonary Tuberculosis. Hosp Pract (Off Ed) (1993) 28:51–8. 10.1080/21548331.1993.11442738 8419415

[155] Cosma CL Sherman DR Ramakrishnan L. The Secret Lives of the Pathogenic Mycobacteria. Annu Rev Microbiol (2003) 57:641–76. 10.1146/annurev.micro.57.030502.091033 14527294

[156] Ernst JD. Macrophage Receptors for *Mycobacterium tuberculosis* . Infect Immun (1998) 66:1277–81. 10.1128/IAI.66.4.1277-1281.1998 PMC108049 9529042

[157] Ramakrishnan L. Revisiting the Role of the Granuloma in Tuberculosis. Nat Rev Immunol (2012) 12:352–66. 10.1038/nri3211 22517424

[158] Marino S Cilfone NA Mattila JT Linderman JJ Flynn JL Kirschner DE. Macrophage Polarization Drives Granuloma Outcome During *Mycobacterium tuberculosis* Infection. Infect Immun (2015) 83:324–38. 10.1128/IAI.02494-14 PMC4288886 25368116

[159] Gideon HP Phuah J Myers AJ Bryson BD Rodgers MA Coleman MT. Variability in Tuberculosis Granuloma T Cell Responses Exists, But a Balance of Pro- and Anti-Inflammatory Cytokines Is Associated With Sterilization. PloS Pathog (2015) 11:e1004603. 10.1371/journal.ppat.1004603 25611466 PMC4303275

[160] Volkman HE Clay H Beery D Chang JC Sherman DR Ramakrishnan L. Tuberculous Granuloma Formation Is Enhanced by a *Mycobacterium* Virulence Determinant. PloS Biol (2004) 2:e367. 10.1371/journal.pbio.0020367 15510227 PMC524251

[161] Huang Z Luo Q Guo Y Chen J Xiong G Peng Y. *Mycobacterium Tuberculosis*-Induced Polarization of Human Macrophage Orchestrates the Formation and Development of Tuberculous Granulomas *In Vitro* . PloS One (2015) 10:e0129744. 10.1371/journal.pone.0129744 26091535 PMC4474964

[162] Tsai MC Chakravarty S Zhu G Xu J Tanaka K Koch C. Characterization of the Tuberculous Granuloma in Murine and Human Lungs: Cellular Composition and Relative Tissue Oxygen Tension. Cell Microbiol (2006) 8:218–32. 10.1111/j.1462-5822.2005.00612.x 16441433

[163] Johansen MD Irving A Montagutelli X Tate MD Rudloff I Nold MF. Animal and Translational Models of SARS-CoV-2 Infection and COVID-19. Mucosal Immunol (2020) 13:877–91. 10.1038/s41385-020-00340-z PMC7439637 32820248

[164] Winkler ES Bailey AL Kafai NM Nair S McCune BT Yu J. SARS-CoV-2 Infection of Human ACE2-Transgenic Mice Causes Severe Lung Inflammation and Impaired Function. Nat Immunol (2020) 21:1327–35. 10.1038/s41590-020-0778-2 PMC7578095 32839612

[165] Chen J Wu H Yu Y Tang N. Pulmonary Alveolar Regeneration in Adult COVID-19 Patients. Cell Res (2020) 30:708–10. 10.1038/s41422-020-0369-7 PMC7338112 32632255

